# The Book World of Medicine and Science

**Published:** 1901-01-05

**Authors:** 


### The Book World of Medicine and Science.

The Mirage of Two Buried Cities. By John Fletcher Horne, M.D., F.R.S.E. (London: Hazell, Watson, and Viney. 1900.)

The two buried cities are of course Herculaneum and Pompeii, and the author's object is to give a presentment of the life, the literature, and the art of these two cities as they existed at the time when they were buried in Vesuvian ashes eighteen hundred years ago. The book is most readable and instructive and is full of interesting details, more especially in regard to the revelations made by the excavators at Pompeii. Under the head of the Professional Life of the Pompeians a description is given of the surgical instruments which were found in what is called the House of the Surgeon at Pompeii, and are now preserved in the Neapolitan Museum. Many of these are said to be "identical" with those in use at the present day. One instrument, indeed—a speculum—is said to be superior, in the author's opinion, to many similar instruments of modern manufacture. Probes, cauteries, lancets, knives, elevators, and catheters, "all give a high idea both of the science and the practice of surgery among the Romans." It would appear, however, that, if we are to judge by the collection of instruments left behind him, the Pompeian surgeon did not aspire to the performance of the major operations of surgery. That, however, seems to us to be a matter of small importance. One might ransack the house of many a practitioner of to-day without finding any indications of the higher surgery, and posterity would get but a poor idea of the operations of the year 1900 if all it had to go upon were the preserved remains of such an establishment. The arts and handicrafts, the social life, the amusements, and many of the customs of the Pompeians are fully dealt with in this book, which is well illustrated with photographs of the various articles referred to, and is altogether a pleasant one to read. No doubt there is a good deal of padding in its pages, but that is inevitable. The author has endeavoured to raise in the minds of his readers a picture—a mirage, as he calls it—of the conditions of life existing at the time when these two cities were buried, and to do this he has had to set the information gained from an investigation of their remains side by side with much that has been obtained from other sources. This work certainly cannot be regarded as the record of a discoverer, but it does give a very good and interesting picture of the times referred to. Which, we take it, is what the author wished it to do.

Who's Who, 1901. An Annual Biographical Dictionary Fifty-third year of issue. (London: Adam and Charles Black. 1901. Price 5s. net).

This year's edition of this indispensable book is enlarged in several directions. The publishers have incorporated with it "Men and Women of the Time," and have added new biographies, including those of all Companions of Orders not previously inserted. Moreover, an attempt has been made to record the relationships of some of the persons whose biographies are given, and thus to add to the interest of the work. In many ways then this volume is better than its predecessors. At this time of day we need hardly sing the praises of "Who's Who," so fully recognised has it become as the universal referee when in doubt as to the constantly-recurring question which its name implies. We may say without hesitation that so often do doubts and queries arise which no other handy volume with which we are acquainted will solve, and so frequently in consequence do we find ourselves referring to it for information, and sometimes even in lazy moments for mere amusement, that we do not know how we should now get on without the familiar "Who's Who" upon our desk. The general information which it contains is well selected; but it is upon its wonderful collection of short biographies that its fame and its utility depend. Anyone can find out something about great and noble personages who occupy high public positions, although even about them some of the details given in this volume might not be easily ascertained on the spur of the moment, but what "Who's Who" tells us in a way that no other book tells, is all about multitudes of people concerning whose very existence the ordinary person may never have heard. There are thousands of such people, occupying high positions in their own spheres—important people in their own circles, literary, artistic, scientific, or what not—yet about whom those who live in other circles may know nothing. The world is too large nowadays to do without "Who's Who."

The Englishwoman's Year Book and Directory, 1901. Third year of new issue. Edited by Emily Janes. (London: Adam and Charles Black. 1901. Price 2s. 6d. net.)

This is a very useful collection of information of many kinds interesting to women. It embraces a wide field, but deals more especially with matters which are so subject to change as to require revision year by year. Thus it is partly historical, partly a directory, partly a guide to women's occupation, and partly an almanac and diary. It is stated in the preface that every paragraph has received careful revision, and that a considerable amount of new material has been added. It is on its frequent and careful revision, so as to keep it constantly up to date, that the real utility of such a work as this depends, and from what we have noticed, this appears to have been well and thoroughly done. Literature, art, athletics, housekeeping, public work, philanthropy, district visiting, clubs, laws affecting women and children, religious work, occupations open to women, are subjects which we pick out at hazard in turning over these all-embracing pages. The amount of information which this Year-Book contains is enormous, and its selections seem on the whole to have been effected with judgment. It certainly is a volume to be recommended for constant reference, and is likely to be especially useful to those women whose interests extend beyond the conventional daily round of household work.

The British Sanatoria Annual. (London: John Bale, Sons, and Danielsson. 1901. Price 2s. 9d. Post free.)

This is a reprint, with additions, of an article which appeared in the "West London Medical Journal" a year or two ago. The additions, however, are numerous, and the illustrations which have been included greatly increase the value of the book. Many details regarding many sanatoria are given, and the issue has been carefully brought up to date. The rapid extension of the hygienic treatment of tuberculosis renders the existence of some such directory as this almost a necessity, and we congratulate the publishers on the manner in which they have produced this really elegant little book.

### NOTES ON BOOKS.

We have received a copy of Herbert Fry's "Royal Guide to London Charities" (Chatto and Windus) for 1900. The preface is not dated, and there is nothing to show to what part of the year, or, indeed, to what year, the statements in the book apply. As a list of charities showing how to direct letters of application or inquiry it, no doubt, serves a purpose, but the information which it contains is of the most meagre description.

